# The forms and purpose of work undertaken by family carers of people living in a care home with a cognitive impairment across a care trajectory

**DOI:** 10.1371/journal.pone.0347638

**Published:** 2026-06-29

**Authors:** Fawn Harrad-Hyde, Christina Faull, Linda Birt

**Affiliations:** 1 LOROS Hospice, Leicester, United Kingdom; 2 University of Leicester, Leicester, United Kingdom; Queensland University of Technology - QUT: Queensland University of Technology, AUSTRALIA

## Abstract

When older people move into care homes, family carers continue to provide unpaid support and care but little is known about how the types of work they undertake and how work changes over time. Drawing on data collected from semi-structured interviews with 29 family carers, we describe six forms of work carers undertook across the care trajectory. These were body work, comfort work, information work, coordination work, safety work and biography work. Whilst the nature of each form of work changed over time, carers undertook work across all phases of the care trajectory. This work was purposeful, undertaken to maintain the care recipient’s dignity, health, wellbeing and functional and cognitive abilities.

## Background and objectives

### Caring for people who live in care homes in England

Caregiving is multifaceted work which can be undertaken in unpaid forms by family and close friends of a person at any stage of their life. It can include providing “physical care, such as bathing [and] feeding a person as well as emotional care, such as tender touch, supportive talk, empathy, and affection” alongside “direct services such as driving someone to a store, or adjusting the medications” (Cancian and Oliker, 2002, p2) [1].

Globally, family carers – such as family members and friends – provide the majority of care for people who are frail, ill or disabled. Approximately 5 million people in England and Wales provide unpaid care [2]. Approximately 59% of carers in England and Wales are women [3]. Women are more likely than men to become carers, provide more hours of unpaid care and provide high-intensity care [3,4]. It is unclear, what proportion of the 5 million carers provide care for people living in their own homes and what proportion do so for people living in care homes.

In the United Kingdom, care homes in each country are governed by different regulatory bodies and policy frameworks. In England approximately 15,000 care homes provide care for approximately 370,000 people [5]. Over half of care home residents are 85 years and older with complex needs such as high levels of physical, functional and cognitive impairment [6]. Care home residents in England are more likely to be older and to have a lower functional status than residents across other European countries [7], yet only one third of homes in England provide nursing services. The large majority of care homes in England are owned by independent companies operating on a private for-profit or private not-for-profit basis [8]. Complexity in care home provision can make selecting a care home challenging for carers.

### The work of family carers across a care trajectory

Across a care trajectory, carers experience a number of transitions. This includes when: the care recipient receives a diagnosis; formal home-care support is sought; and when the person moves into a care home [9]. At the turn of the twenty-first century, researchers began to describe carers’ experiences of providing care before, during and after the move into a care home [10–12]. These studies “helped debunk the myth that carers abandon their relatives in nursing homes or similar settings” (Gaugler, 2005, p105) [13], yet highlighted that for most carers, care homes were “strange and alien environments...of which they had little previous experience” (Davies and Nolan, 2004, p520) [10]. Although these studies provided insights into the experiences of carers across transitions in place of care, to date little is known about the forms of work carers undertake and how work may change across transitions.

The word ‘work’ is often used to denote paid employment but may encompass activities that are unpaid [14]. The work of (unpaid) carers has largely been invisible. Friedemann-Sánches and Griffin (2011) suggested this is, in part, due to scholars using the terms ‘unpaid caregiving’ and ‘unpaid labour’ interchangeably [15]. Friedemann-Sánchez and Griffin (2011) differentiated between ‘unpaid labour’ as activities to support people who were able to look after themselves and ‘unpaid caregiving’ as activities to support people who were dependant (for example due to age or illness). They argued that unpaid caregiving is distinct from unpaid labour in several ways. Unpaid caregiving is emotionally laden, relational and influenced by the characteristics of the caregiver, care recipient and the relationship between the two [15].

The conceptualisation of unpaid caregiving as gendered work gained traction during the 1970s, when scholars sought to highlight the unpaid work of women within the home [16,17]. This continued during the turn of the century, with scholars arguing that unpaid work was of equal importance and value to paid employment [18,19]. Prior to this, caregiving was recognised as neither skilled, nor as a form of work within policy, and carers were predominantly seen to be women, looking after others within a home environment [20]. More recently, research has explored cultural experiences of caregiving [21]. Whilst familial, cultural, religious values can shape individual experiences of care-giving, there are also shared values that are common across cultures, including values related to love, reciprocity and responsibility [22].

Scholars have also applied concepts from studies of paid labour to unpaid labour, including caregiving. This work has focussed on ‘caregiver strain’, demonstrating the burdens associated with caregiving – including the impact on physical health, mental health, economic position – and the difficulties individuals face when balancing caring alongside existing hobbies, relationships and paid work [23].

Our paper focusses on carers who supported a person living with a cognitive impairment in a care home in England. It explores the different forms of work carers undertook across a care trajectory, including when the person they cared for lived at home, during and after the move into a care home and at times of deterioration and end-of-life. The term ‘care trajectory’ is used deliberately, akin to the way Strauss and colleagues (1985) describe an ‘illness trajectory’ as encompassing “*not only the physiological unfolding of a patient’s disease but the total organization of work done over that course, plus the impact on those involved with that work and it’s organisation*” (Strauss et al, 1985, p8) [24]. This can include the work of people receiving care and those who support them, either through paid employment or unpaid caregiving. Each illness trajectory has a number of ‘trajectory phases’ and across a trajectory individuals may undertake a variety of forms of work.

Strauss and colleagues (1985) outlined five types of work carried out across an illness trajectory. ‘Machine work’ included activities undertaken to set-up and monitor machines, and to connect machines to patients’ bodies. ‘Safety work’ described activities to assess, prevent and rectify risks associated with medical interventions. ‘Comfort work’ outlined activities to assess, prevent and minimise the physical discomforts associated with medical care, for example pain and nausea. ‘Sentimental work’ included work to provide emotional support to others and ‘articulation work’ described activities to oversee the trajectory, including anticipating the potential directions a trajectory may take and potential risks that may occur. In addition, Strauss and colleagues (1985) discussed their findings in relation to four existing theoretical concepts. These were: ‘error work’ to define and rectify mistakes; ‘dirty work’, which included tasks which were actually or symbolically dirty; ‘information work’, to gather, manage and communicate information; and ‘body work’, which included working with patients’ bodies. Corbin and Strauss (1985) also used the concept of an illness trajectory to explore the work of patients and families managing chronic illnesses at home alongside their everyday lives [25]. They suggested patients and families undertake: ‘illness work’ to manage the person’s illness; ‘everyday work’ to maintain existing responsibilities such as home-making and childcare; and ‘biographical work’ to maintain a coherent life narrative. Corbin and Strauss (1985) highlighted the ways individuals balance different forms of work, suggesting that during changes in trajectory phases, people have to establish new routines of work to maintain balance.

Subsequent research further developed conceptual understandings of the work of carers. The concept of biography work has been applied to individuals who experience an illness [26] and those who support them [27]. In addition, the concept of ‘biographical disruption’ [28] has been used to explore the impact of receiving an illness diagnosis – both on the person [26] and on others [29]. The term ‘dirty work’ has been applied to caregiving tasks that involve bodies and their associated discharges and dysfunctions [30,31], yet some authors have called for ‘dirty work’ to be reconceptualised as ‘dignity work’ [32,33] to reflect that undertaking this type of work can provide the caregiver with a sense of pride and maintain a care recipient’s dignity.

Strauss and colleagues’ (1985) description of ‘sentimental work’ overlaps with the concept of ‘emotion work’, described by Hochschild (197) as work undertaken to manage or conceal one’s emotions in order to maintain relationships [34]. The concept was further developed by to emphasise this form of work is often undertaken to enhance another person’s well-being [35]. Cares often engage in emotion work [36]. For example, carers of people living with dementia may conceal their negative internal emotions (such as sadness or frustration) to present emotional responses that are more beneficial for the care recipient [37,38]. Carers may also conceal the needs of the person, the level of care they provide and the burdens they experience to ‘save face’ and avoid burdening others [39].

In addition to studies focussed on a specific type of work, empirical studies have continued to underline the multifaceted nature of caregiving, describing ways carers continue to provide support once the care recipient moves into a care home. Streeter (2023) suggested families undertake activities to: ‘personalise care’, by labelling clothes, decorating rooms and bringing in preferred food and drink items; ‘coordinate care’, particularly in relation to external services such as chiropodists; and to ‘advocate for change’ by making suggestions for improvement. At times of deterioration in a person’s health, carers may contribute to decisions about care and treatment [40], and act as an ‘advocate’ or ‘spokesperson’, especially when the person has a cognitive impairment [41]. However, carers can find decisions-making challenging when they are unsure of the person’s wishes or when they feel unsupported [42].

Contextual factors shape the involvement of carers in care homes. In care homes with lower staffing levels, carers may be expected to ‘plug the gaps’ in care, whereas, in care homes with higher staffing levels, carers may only be expected to socialise with residents [43]. As such, there have been calls to refer to carers of care home residents as ‘family workers’ to acknowledge the time, skills and work required [40].

The aforementioned studies have uncovered different forms of work undertaken by carers. Whilst some have focused solely on one form of work, others have described carers’ experiences and the activities they undertake. However there has been limited consideration of how forms of work change over time. In this paper we outline the different forms of work undertaken by carers across a care trajectory.

## Research design and methods

This aim of this paper is to describe and examine the different forms of work undertaken by family carers of people who live with a cognitive impairment in a care home, and to consider how the work of carers may change as they support a person across a care trajectory – including caring for a person living at home, during and after the move into a care home and at times of deterioration and end-of-life. The data presented were collected as part of a study which explored the potential role for peer-mentors to support carers to prepare for discussions and decisions about deterioration and end-of-life [44]. Ethical approval was obtained from the University of Leicester’s Research Ethics Committee for Medicine and Biological Sciences (Reference: 32446).

The study design was guided by the philosophical assumptions of social constructionism, which suggests there is no single reality. Rather, people construct meanings grounded in personal and cultural contexts [45]. In line with this approach, rather than seeking to describe a universal static ‘reality’ that is ‘true’ for all carers, we accepted that different participants may present diverse and divergent experiences of the work they undertook in their caring role.

### Reflexivity and positionality

All three authors had experience of being a carer and supporting someone to move into a care home. Constructionist approaches to data collection encourage researchers to draw on their own experiences to make sense of data [46]. However, to enhance dependability of interpretations, summaries of emerging findings and initial themes were discussed amongst the wider research group and with two stakeholder groups. The first stakeholder group consisted of nine people with lived experience of supporting a person with a cognitive impairment in a care home. The second stakeholder group consisted of approximately 15 people, including carers and representatives of local organisations that support carers. Five meetings were held with each group. During the meetings, stakeholders provided input by discussing the development and appropriateness of study materials, reviewing recruitment progress and suggesting potential strategies and reflecting on emerging themes and findings. At each meeting, either FHH or LB made hand-written notes which were later typed and circulated to group members to ensure attendees agreed with the account recorded. These notes were referred to at later stages of analysis and during the writing of subsequent outputs.

### Setting, sampling and recruitment

People were eligible to participate in the study providing they were either a current or bereaved carer (within the previous 36 months) of a person living with cognitive impairment who lived in a care home in England. Data extracts for current family carer and bereaved family carer participants are labelled using ‘FC’ and BC’ respectively. The study was advertised widely via social media platforms, hospice newsletters, and the research teams’ and stakeholder group members’ networks. Posters were also placed in communal areas of local care homes across Leicester, Leicestershire and Rutland. Potential participants self-identified by expressing an interest in the study by email or telephone. As recruitment progressed, the research team reviewed participant characteristics and used purposive sampling to select carers who were: different ages, ethnicities and genders, who had been caring for their relative for different periods of time and who had different forms of relationships with a person residing in a care home.

### Procedures

Once people expressed an interest in the study, FHH or LB contacted the potential participant to confirm eligibility criteria. Eligible individuals were posted or emailed a study information sheet. Follow-up contact was made to address further questions. If potential participants did not respond to two research team contacts, they were considered to not want to participate. Participants took part in a semi-structured interview between 21/03/22 and 27/04/23. Given the timing of the study and the requirement for participants to have had experiences of supporting someone who lived in a care home within the previous 36 months, many participants had experiences of supporting someone whose care trajectory overlapped with the Covid-19 pandemic.

Interview schedules were developed through a review of existing literature and through stakeholder engagement. Interviews were predominantly undertaken by FHH, with LB undertaking two interviews. Both interviewers were female. Interviews were audio-recorded and transcribed verbatim. During the transcription process identifiable data was removed and each participant was allocated a participant number. Interviews lasted between 45 and 85 minutes (averaging 63 minutes) and took place either face-to-face (in the participant’s home or researcher’s workplace) or virtually via Microsoft Teams^©^. Data collection occurred in parallel with analysis and continued until the team made a judgement that theoretical saturation had been achieved and that further data collection was unlikely to result in additional themes [47].

### Analysis

Data were analysed thematically, in line with Braun and Clarke’s reflexive thematic analysis approach, in order to generate contextualised themes [48]. This approach can be applied across epistemological approaches. In this study, a constructivist approach was adopted to allow for diverging experiences to be presented [49]. Each transcript was printed, read and re-read to enable the team to become familiar with the data. Then, each transcript was hand-coded on a line-by-line basis according to the phenomenon or concept that was being discussed – thus generating a number of open codes. Following hand-coding, NVivo was used to manage data and further explore relationships between codes. Then, the relationships between codes were considered, allowing for the generation of initial themes. FHH coded all transcripts and LB independently coded five transcripts. Consistent with a constructivist approach, FHH and LB met to identify and discuss similarities and differences regarding the ways in which each respective author had interpreted the data to produce codes and themes and to consider how the emerging findings related to established literature. Doing so enabled the authors to refine, re-organise and name final themes, presented below.

## Results

Twenty-nine family carers of people living with a cognitive impairment in a care home were interviewed (23 female, 14 currently caregiving). Twenty-four supported a person living with dementia. The remaining five supported a person who had cognitive impairments due to stroke (three people), acquired brain injury (one person) or global developmental delay (one person). Nineteen supported a parent, five supported a spouse and five supported another relative (see Table 1 for further information).

**Table 1:** Demographic characteristics of participants.

Variable	n	%	Mean	Range
**Status**				
Current family member	14	48		
Bereaved family member	15	52		
**Age (years)**			57.51	27-91
20-30	1	3		
31-40	1	3		
41-50	7	24		
51-60	11	38		
61-70	4	14		
71-80	3	10		
81-90	1	3		
91-100	1	3		
**Gender**				
Female	23	79		
Male	6	21		
**Ethnicity**				
White British	21	72		
Asian British	3	10		
White Irish	2	7		
Mixed White and Asian	1	3		
Mixed Chinese	1	3		
Any mixed other	1	3		
**Caring for**				
Parent/ parent-in-law	19	65		
Spouse	5	17		
Other	5	17		

Six forms of work are described and examined. These are body work, comfort work, safety work, information work, coordination work and biography work. Before outlining the different forms of work, it is important to contextualise our interpretations. Whilst some participants referred to the support they provided as a form of ‘work’, others did not use this language, instead discussing the support provided as rooted within familial bonds or cultural expectations surrounding caring for others. Many participants juggled caregiving work alongside paid employment and additional unpaid caring responsibilities, often whilst also dealing with other significant life events, including bereavements and serious health concerns.

Whilst most participants provided care in-person, others provided and coordinated care remotely. Some participants made significant adjustments to their lives to accommodate their caring work (such as reducing or stopping paid employment) and some received support from formal domiciliary services. Despite this, the majority of participants described reaching a *“breaking point”* (FC09), at which caring for the person at home was no longer a viable option. This appeared to be a catalyst, which led to discussions about whether the person may need to live in a care home.

*My sister and I were literally sleeping in the same room with her at night. And 24/7 care kills you… We were suffering. (FC02)*

Following the move into a care home, the presence of a team of paid staff had the potential to reduce the frequency and intensity of many forms of work undertaken by participants. This had the potential to reduce some of the responsibilities placed upon them. However, participants continued to undertake diverse forms of work.

*At home it was almost like a full-time job… but then every time you go into the care home, there’s an issue…it just sort of never stops! (FC10)*

Occasionally participants undertook a substantial amount of work, beyond what they expected when the person moved into the care home. Participants reported that in these circumstances the work felt very difficult, as they continued to hold the ‘weight’ of responsibility for the person living in the care home.

*I didn’t think there would be no support, no nursing component… I felt like we had that responsibility to take care of Mum. We were there around the clock. (FC12)*

Finally, whilst our presentation of different trajectory phases may appear linear, eight participants described complex trajectories in which participants moved *between* different care homes. Doing so opened up the possibility that carers could become involved in cyclical work, for example having to search for and support a person to move into a care home, only to later repeat this process in the event the person moved to another home. Below, each of the six themes are reported.

### Body work

Participants carried out ‘body work’, which involved performing tasks involving the care recipient’s body. Many participants provided assistance with personal care or helped the person to eat and drink and to move and transfer. This work was described as physically demanding and difficult to manage.

*Getting him onto the [mobility equipment] and the transferring him from the [equipment] onto the bed… he just sort of slid between the [equipment] and the bed at one stage! (BF11)*

Participants described different levels of ease in undertaking this form of work. For example, two male participants caring for older females, suggested undertaking body work could feel uncomfortable.

*For a man in my culture to have to do it and look after a woman, especially when they need cream in between their thighs, unfortunately I had to do that, which was so disrespectful - not only to me but to her. (BC05)*

Once the person moved into a care home, participants undertook fewer body work tasks. However, some undertook specific tasks such as supporting the person to wash, brush their hair or teeth. Participants were more likely to undertake these tasks if they perceived the care home staff had not completed the task, or not completed it to a satisfactory level. Similarly, at times of dying and deterioration, some undertook specific tasks, such as mouth care.

### Comfort work

Participants also undertook ‘comfort work’ aimed at ensuring the person felt physically and psychologically comfortable. Whilst the person lived in their own home, participants undertook domestic tasks. These tasks could be considered practical tasks, however performing them often aligned with the goal of ensuing physical or psychological comfort. For example, this could include cleaning (to ensure the environment was physically comfortable), and shopping for food and drinks (so the person would not be hungry or thirsty) and appropriate toiletries (to enable them to be and feel physically clean). Participants also provided social support and companionship. Then, once the person had moved into the care home, some participants described accompanying the person to activities within the home, or taking the person out of the care home for social activities. Some participants also provided support and reassurance to the person at times of distress. For example, some discussed comforting the care recipient if they experienced distress at the point of a care transition, and a further two participants discussed being called in to the home at times the care recipient experienced distress. On both of these occasions the distress was experienced by a person living with dementia.

*I was at work and she was having one of these episodes and [the care home staff] rang me and said ‘you need to come now and calm your mum down - she’s shouting”… I got there and sat with her for a good couple of hours. (BFC14).*

Whilst looking for a care home, participants considered whether the person may *feel* physically and psychologically comfortable within a particular home. Then, once the person moved into a care home, participants talked about the shift in the work they undertook, from personally maintaining the person’s comfort to monitoring both the person (for example, looking for signs the person was in pain) and care home environment and raising any concerns they had.

*Our role became more of a supportive role… monitoring his room in the home, keeping check of everything, his wellbeing and his health in there. (FC04)*

Other participants sought to ensure the person felt comfortable by creating a sense of normalcy within the care home. On admission, participants personalised the person’s room, or provided information to support staff to do so. Participants brought in items that held sentimental value or could provide entertainment, such as televisions, radios and books. Participants also attempted to create routines for the person.

At times of dying and deterioration, participants continued to work to ensure the person was physically and psychologically comfortable. For some, this was achieved by visiting or ‘being with’ the person to bring psychological comfort. One participant said *“I just hold his hand and hope he feels loved”* (FC08). For others, comfort work could include attempts to ensure the person was physically comfortable: *“I wanted to put a pillow between his head and the rail. I didn’t feel he was comfortable”* (BC11).

### Safety work

Participants discussed a range of ‘safety work’ activities undertaken to ensure the physical safety of the person. Whilst the person lived at home, participants worked to prevent falls and accidents. Some used forms of technology, for example, one participant said: *“He’s started wandering out at night so we’d put whiteboard up saying ‘stay in your flat’”* (BC07). Sometimes, concerns about the person’s safety contributed heavily towards the decision to move into a care home.

Once the person moved into a care home, including at times of deterioration, participants talked about a change in the work they undertook. As with aspects of comfort work, rather than being directly responsible for doing safety work, participants talked of “monitoring” the person and the environment to ensure that *“things had been done properly”* (FC06). However, doing so was not without challenge. Two participants feared that raising concerns may have negative repercussions for the person living in the care home. The ability to monitor safety was also particularly difficult during periods that visiting was restricted, such as during the Covid-19 pandemic. This, in turn, had the potential to inhibit the development of trustful relationships with care home staff:

*We always had to visit in the conservatory… I felt as though they were hiding something… I didn’t know what was going on behind closed doors. (BC10)*

### Information work

Participants undertook ‘information work’ to gather, understand and share information required to enable themselves and others to support the person. During early phases in the care trajectory, participants learned about the causes, symptoms and difficulties related to the person’s health conditions and cognitive impairment. They did so by talking to others, attending group sessions and reading leaflets and online materials. At later phases of the care trajectory, participants sought information regarding palliative and end-of-life care.

Whilst looking for a care home, participants undertook a substantial amount of information work, drawing upon several sources including personal connections, charitable-, third- and voluntary- sector organisations, social services, healthcare professionals and online information. Participants described things that complicated this information work. These were: a lack of understanding about the care home sector, such as different types of care homes and funding; feeling ‘rushed’ into selecting a care home, either because they had not explored care home options until they reached a ‘breaking point’ or because the person was awaiting a hospital discharge; a perceived lack of support available for people who were ‘self-funding’ their care; and the impact of the Covid-19 pandemic, which limited opportunities to visit homes.

*I felt out of my depth completely… You’re sort of free-falling in the dark. (BC03)*

Participants also discussed the work involved in communicating with the person, for example, altering their communication style to accommodate the person’s cognitive impairment and determining how to share information with them. After the person had died, participants continued to undertake information work, informing family members, registering the person’s death and organising post-death rituals.

### Coordination work

Participants undertook coordination work, overseeing the overall arc of work that occurred across the care trajectory. Whilst the person was living at home, participants managed the person’s medications and organised and attended health and social care appointments. This required participants to liaise with various health and social care professionals, which was something that continued during the search for an appropriate care home.

Once the person moved into the care home, participants continued to coordinate the person’s care, for example by supporting the person to access services that were not routinely available in the care home and accompanying the person to external appointments. Some suggested their level of coordination work reduced when the person moved into a care home. Reflecting on the change, one participant stated: *“I wasn’t a carer anymore, I was like an admin ancillary person”* (BC13). Many described this as a welcome change, that enabled them to “*step back and be family again”* (BC01), facilitating a move towards the relationship that existed before the person needed additional support. However, some acknowledged the consequences of this change:

*Sometimes things would happen, like a call to a GP or change in medication, and I wouldn’t necessarily know, which I would have done before. (BC02)*

However, especially during transitional periods such as when a care recipient’s needs changed, some participants continued to undertake coordination work.

*They were saying she needed a nursing home to cope with her needs… the stress on me trying to coordinate all these people to get them to do what needed to be done. (FC10)*

Much of the coordination work focussed on issues the carer and care recipient faced in the present. Only a small minority of carers suggested they had spent time anticipating future issues that could occur, with one participant stating *“we never had time for that”* (BC03).

At times of deterioration and end-of-life, participants continued to undertake coordination work, for example through involvement in decision-making. Participants described varied levels of involvement: some contributed to written documents and care plans, some were involved in decisions at the time of deterioration, yet others said they had not been involved in discussions or decisions. Participants described instances when they had to negotiate with others about how best to respond to a deterioration and to ensure people involved were *“on the same page”* (FC14) and aware of the person’s stated wishes and/or written plans. One participant stated they had to be: *“fairly firm with professionals… to defend [their] elderly relative against the disruption of going into hospital”* (BC02). Another described navigating such tensions as “*hard work” (B15).*

### Biography work

Participants undertook ‘biography work’ to ensure the person’s broader life story was maintained, understood and respected. Whilst the person was living in their own home, participants encouraged the person to maintain pre-existing hobbies, interests and friendships. Whilst looking for a care home, as well as considering whether a home could accommodate the person’s needs, participants considered whether the care home might be a ‘good fit’ for the person – both in the present and across the imagined future care trajectory.

*Dementia is progressive, the places that might be right for somebody with severe dementia aren’t for somebody with milder dementia and it was quite difficult to find a fit. (FC02)*

Once the person was in the care home, participants worked to ensure staff understood the person’s life story. Whilst most of this work was completed on admission, participants described on-going work to contextualise the behaviours of the person, to ensure the staff and sometimes other residents understood the person:

*Staff were trying to suss out her behaviour – they’d test the fire alarm and Mum would leap out of her seat… I said “she worked in a school so if the bell went that’s what she would be doing”. (FC10)*

Participants’ biography work intersected with other forms of work. For example, this work intersected with coordination work to ensure everyone was aware of the person’s wishes, for example at times of deterioration and end-of-life. In addition, the desire to create a sense of normalcy by creating an environment with familiar objects and routines – discussed earlier as a form of comfort work – intersected with the desire to affirm and respect the previous identity of the person. One participant made explicit links between the desire to maintain a sense of normalcy and continuity in how the person looked and dressed as a means through which to maintain their dignity and identify:

*I went to visit mum and she was sat in the lounge in her nightie, a jumper, her outside coat, slippers and handbag… When I got home, I cried. I felt embarrassed for mum… because she was a proud lady and she was super smart. (FC05)*

### Caregiving work as purposeful

A common feature of the work undertaken by carers across the care trajectory was that was it was purposeful, often aimed at achieving particular goals. This was evident in one of the extracts above, in which the participant described undertaking biography work to ensure both care home staff and other residents understood the persons current behaviour in the context of their broader biography. Such work was undertaken to ensure the care recipient’s identity and dignity was maintained. In addition, one participant described working to achieve a sense of normalcy, by creating routines, to encourage the person to eat, recognising that this, in turn, could support their health and wellbeing.

*Whenever I went to speak with her, I coaxed her into eating… I tried to make things look very normal and give her a proper routine. (BC05)*

Similarly, when talking about providing social support and activities, participants noted that as well as being motivated by a desire to spend time with the person, they were also trying to preserve the person’s physical and cognitive abilities.

*I try to keep her occupied… I bring in games and puzzles… I fear that she would lose the ability. You don’t use it, you lose it. (FC07)*

The findings that carers undertake different forms of work to achieve particular goals could also be seen at times of deterioration. Participants sought to advocate for a person’s wishes surrounding deterioration and end-of-life to ensure the person received care and support that aligned with their wishes and best interests.

*The ambulance turned up and were going to take her to hospital… It was a battle. Nobody talked about moving the Queen to hospital did they - why are we moving my mother?! (FC14)*

## Discussion and implications

Our findings offer new understandings of the different forms of work that family carers undertake whilst supporting a person with cognitive impairment at various phases of a care trajectory – including before, during and after a move into a care home and at times of deterioration and end-of-life. This study has described six forms of work that carers undertake – body work, comfort work, safety work, information work, coordination work and biography work. Situating the data within the theoretical conceptualisation of work and using the theoretical lens of a ‘care trajectory’ has provided an interpretive framework through which we have been able to explore the ways this work may change across a care trajectory. In doing so, we have been able to demonstrate that whilst the specific nature of work may differ across people and may change, transform and fluctuate across time, carers undertake work at all phases of the care trajectory. Furthermore, our study has highlighted that the work undertaken is purposeful, and that carers undertake this work to maintain the person’s dignity, health, wellbeing and functional and cognitive abilities.

### Comparison with theoretical literature

Several of the forms of work have been described within existing literature. For example, activities involving the bodies of others has been described as both ‘dirty work’ [31], and ‘dignity work’ [32,33]. We use the neutral term ‘body work’, but highlight the ways an individuals’ demographic characteristics and culture can shape their framing of this work. Further research could employ an intersectional approach to understanding people’s experiences of providing body work.

The terms ‘information work’ and ‘safety work’ are used here in line with Strauss and colleagues (1985) [24]. We have also used the term ‘biography work’ akin to the description by Corbin and Strauss (1985) as work to maintain a coherent life narrative [25]. In contrast to existing research which focusses on the ways individuals maintain a coherent life narrative for themselves, our research suggests family carers also do work to maintain a coherent life narrative for the care recipient. Whilst Strauss and colleagues (1985) differentiated between ‘comfort work’ to prevent the physical discomforts and ‘sentimental work’ to provide emotional support, our paper conceptualises ‘comfort work’ as including activities to provide both physical and psychological comfort.

Our conceptualisation of ‘coordination work’ overlaps with Strauss and colleagues' (1985) notion of ‘articulation work’, however we have selected the term coordination work to reflect activities undertaken to ensure various different people were *‘on the same page’*. Whereas ‘articulation work’ involves envisioning potential courses that an overall trajectory may take, including potential risks a person may have and potential ways to overcome these, the ‘coordination work’ described by our participants appeared to be predominantly present-, rather than future-focussed.

There are two notable absences within our data. The first is Strauss and colleagues’ (1985) notion of ‘machine work’. Whilst some participants talked about using equipment, such as aids to support a person to mobilise, these were not a prominent feature in participants’ responses. The second notable absence is that whilst participants discussed a range of feelings associated with caregiving, they did not report undertaking ‘emotion work’ [34]. It is likely carers do undertake this form of work, and there are several possible explanations for this absence. Firstly, it may be that the aim of the broader study and the questions asked – which explored the potential role for peer-mentors to support carers to prepare for discussions and decisions about deterioration and end-of-life [44] – did not provide sufficient opportunity to surface discussions related to emotion work. Second, set against a backdrop of health and social care services in which care is often conceptualised as a series of tasks to be performed [50,51], it is possible that participants may have considered this form or work, which is often invisible, to be less valuable than other forms of work. Thirdly, it is possible that participants viewed emotion work as work that is performed privately, which may have reduced the likelihood they would discuss this during an interview. Future research could explore the forms of work that are absent in the current paper. In addition, given that participants used metaphors, such as “free-falling in the dark” to describe their experiences, future work could utilise such metaphors during data collection to prompt discussion of emotion work [52], although cross-cultural understandings of such metaphors need to be carefully considered.

As noted earlier, many participants in our study were juggling caregiving work alongside paid employment, additional unpaid caring responsibilities and significant life events. This finding echoes a large body of evidence which has highlighted complex issues carers face when attempting to remain in the workforce or to re-enter the workforce after a period of caring [53–55]. Whilst it is possible that combining caring work and paid employment can personally rewarding for individuals – and paid employment can provide a respite from caring – overall, published research has suggested caregiving can have a negative impact on an individual’s work-life [56]. The use of the ‘care trajectory’ [24,25] concept could provide a theoretical lens through which to further explore the ways changes in one form of work (such as the work involved in caregiving, ‘everyday work’ or ‘paid employment’) may impact on other forms of work.

### Comparison with applied literature

The results presented add to the wider knowledge base surrounding family carers’ experiences during transitions in care, which have been explored in applied, empirical studies [10–12]. Our participants often experienced the work of providing care in a person’s own home as burdensome and beyond their capabilities. This echoes the work of Samsi, Cole and Manthorpe (2022) who suggest carers may reach a ‘tipping point’, exhausting all other options prior to considering a move into a care home [57]. However, once carers began looking for a care home they often struggled to know where to look for information and how to interpret information regarding care homes. Many participants found it challenging to combine paid employment with their unpaid caregiving work. This finding provides much needed contextual information to existing research which has suggested carers are less likely than the general population to be in work. Further research could focus on carers’ decisions to leave paid employment and on identifying factors that enable carers to remain in employment.

### Contextual influences on the work of family carers across a care trajectory

The findings presented also demonstrate the impact of contextual factors on the work of family carers. For example, whilst COVID-19 had a devastating impact on those who lived and worked in care homes [58,59] the findings presented suggest the pandemic added a layer of complexity to the work of carers at various phases of the care trajectory. Carers found it difficult to identify a suitable care home without opportunities to visit and once a home was found, restrictions surrounding visiting limited the ability for carers to develop trustful working relationships with staff [60]. Furthermore, work undertaken by carers appeared to shaped by perceptions of the level of care provided in the care home, especially if carers had concerns that care home staff may not be undertaking these roles. This supports Lowndes et al.’s (2023) suggestion that carers ‘plug the gaps’ in care, undertaking work care home staff do not have the capacity to fill [43].

Whilst a person’s move into a care home had the potential to reduce the frequency and intensity of many forms of work undertaken by participants, some continued to undertake a substantial amount of work. This was experienced as problematic when the work undertaken did not match participants’ expectations of what they had anticipated doing once the person moved into the home. As population-level demographics shift and more people reach the end of their lives in a care setting, further research is needed to explore and understand differences in expectations of care between carers and care home staff and, importantly, care recipients.

### Implications for practice

The work of family carers can often be invisible and unrecognised by the formal health and social care services that carers and care recipients interact with. This invisibility may be heightened for carers of people who live in a care home environment, due to an assumption that once a person moves into a care home, carers are no longer required to complete the work that they were previously undertaking. Our findings challenge this assumption and provide evidence to demonstrate that although the work undertaken by carers may change over time, they are undertaking several different forms of work across all phases of a care trajectory.

Our finding, that the coordination work undertaken by carers was largely present- rather than future-focussed, supports previous research which has suggested people living with dementia and their family carers recognise their needs will change over time, but uncertainties about the degree of change and the timescale of changes prevents them from planning for these changes [61]. This finding has implications for policy and future work is needed to explore ways to encourage and support people living with cognitive impairment and their family carers to think about future care needs.

Across the different forms of work undertaken by carers in our study, participants described undertaking skilled care tasks without training or guidance. This included supporting someone to manoeuvre safely or adjusting communication styles to accommodate a person’s cognitive impairment. Previous research has called for families of care home residents to be referred to as ‘family workers’, to acknowledge the work they undertake and to facilitate conversations that promote safe ‘working’ conditions for them [40]. Our findings suggest that a broader consideration of the work that families do across the care trajectory, the conditions under which they work, and how to provide appropriate training and education for family carers, could be beneficial to both carers and care recipients.

### Strengths and limitations

The strengths of the study include strong stakeholder engagement throughout. Accounts from current and bereaved participants reduced the risk of only hearing accounts of caring that have been ‘diffused’ over time [62]. Participants were predominantly female, yet the findings capture a diverse range of experiences of male carers, including sole male carers (supporting a spouse or an aunt) and a male carer supporting a parent-in-law alongside their female spouse. Whilst there is limited ethnic diversity in the sample, the research team deliberately sought to increase diversity by advertising the study widely and seeking participants that could be under-represented, for example people from ethnically diverse backgrounds.

The focus of the larger study for which this data was collected may not have enabled the research team to capture an exhaustive list of all the different forms of work that participants undertook. As has been described earlier, two notable absences within our data are ‘machine work’ and ‘emotion work’. Furthermore, our data was collected at a single-point in time. Future work could use a longitudinal approach to further explore how the work of carers changes over time across a care trajectory.

This study aimed to describe the different forms of work undertaken by family carers, by gathering data from family carers directly. In doing so, we have not captured the views of care recipients. Further work could gather data from people receiving care to explore their views on the work undertaken by family carers. Similarly, within this study, we focussed on the work undertaken by family who supported a person with a cognitive impairment who had moved into a care home. Further work could explore and compare the potential differences in the work undertaken by different groups carers, such as carers of people with and without a cognitive impairment, carers of people who do and do not live in a care home or carers of different demographic characteristics.

## Conclusion

Carers of people living with a cognitive impairment in a care home provide substantial support at all phases of the care trajectory – including whilst the person is living at home, during and after a move into a care home and during deterioration and end-of-life. Providing support requires family carers to undertake various forms of work. This includes body work, comfort work, safety work, information work, coordination work and biography work. Whilst the specific forms of work undertaken may vary between people and across time, carers undertake work at all phases of the care trajectory. This work is purposeful, often performed to maintain the person’s dignity, health, wellbeing and functional and cognitive abilities.
