# Supplementary material for: The forms and purpose of work undertaken by family carers of people living in a care home with a cognitive impairment across a care trajectory
Source: PLoS One. 2026 Jun 29;21(6):e0347638. doi: 10.1371/journal.pone.0347638 (PMC13313323; doi:10.1371/journal.pone.0347638)
Supplement: Attachment — Submitted filename: Response to Reviewers.docx [file pone.0347638.s001.docx]

**Response to Reviewers**

Manuscript: PONE-D-25-62443 - The forms and purpose of work undertaken by family carers of people living in a care home with a cognitive impairment across a care trajectory

06/03/2026

Dear Editor,

We are pleased to re-submit our manuscript titled: “The forms and purpose of work undertaken by family carers of people living in a care home with a cognitive impairment across a care trajectory” (reference: PONE-D-25-62443).

We would like to thank our reviewer for taking the time to read and review our paper. We are grateful that they acknowledge the paper’s strengths, alongside areas which could be further improved. Below, we outline how we have addressed each of these comments on a point-by-point basis. In several of the points we have changed the wording in our manuscript to align with the reviewer comments. New additions to existing sentences have been marked in **bold** font.

There are two related reviewer comments that we do not feel able to action and we feel it is important to provide sufficient information to explain why we are not able to do so. Both of these comments ask for us to provide a more nuanced discussion around the ways in which family carers’ culture may influence their experience of undertaking care work. We have revisited our manuscript and our data in order to carefully consider the reviewer’s comment. Within our manuscript, we currently raise the issue of culture, briefly, on two occasions:

1. We have a verbatim quote under the theme ‘Body work’, in which a participant said: “For a man in my culture to have to do it and look after a woman, especially when they need cream in between their thighs, unfortunately I had to do that, which was so disrespectful - not only to me but to her.”
2. We have a sentence in the discussion (which the reviewer quotes), which reads: “We use the neutral term ‘body work’, but highlight the ways an individuals’ demographic characteristics and culture can shape their framing of this work.”

Whilst we fully agree with the reviewer that culture is likely to impact on carers’ experience of providing care and undertaking different forms of work, we are not able to expand on this point as this was not a specific focus of the research and we have insufficient reference(s) to culture in our data; hence we are not able to action these two comments.

Please note the line numbers listed in the table below relate to the line numbers in the document titled ‘Revised Manuscript with Track Changes’.

Thank you again for considering our manuscript. We look forward to your response and are happy to provide any additional information required.

Sincerely,
Dr Fawn Harrad-Hyde (BSc, MPhil, PhD)
[fawn.harradhyde@leicester.ac.uk](mailto:fawn.harradhyde@leicester.ac.uk)
On behalf of all co-authors: Dr Linda Birt and Professor Christina Faull

General comments:

| Reviewer comment: | Response: | Location: |
| --- | --- | --- |
| I did think that the authors’ reference to ‘accuracy’ (line 199) is perhaps inconsistent with their social constructivist philosophy – do they mean here that they checked back with stakeholders for their agreement on the account recorded? | We have now amended this line to remove the term ‘accuracy’:  At each meeting, either FHH or LB made hand-written notes which were later typed and circulated to group members **to ensure attendees agreed with the account recorded**. | Lines 201-204 |
| The methods are well described, in particular the approach to recruitment and purposive sampling. I personally feel it is a shame that these were just one-off interviews, as longitudinal interviews would have given more opportunity to explore change over time, but focusing on carers’ accounts on trajectories is still valuable. This could perhaps be picked up in the limitation section. | Thank you, we agree that a longitudinal approach would be useful and have added in the following sentence to the limitations section:  Our data was collected at a single-point in time. Future work could use a longitudinal approach to further explore how the work of carers changes over time across a care trajectory. | Line 675-679 |
| I would like to see a little more detail in the Analysis section. In particular, how did the constructivist approach influence analysis? How did you move from codes to themes? Were stakeholders involved in this process? | We have now included more detail in this section to address these comments. | Lines 242-257 |
| The role of culture in care work is raised a few times in the Findings and I think this was an omission from the Background section (for example, there was nothing about how carers in different cultures experience care work). Similarly, the influence of culture on experiences of care could be returned to in the Discussion section. | We agree culture is important, however there is insufficient data on cultural differences to explore this in more detail. Therefore, we have not added to the discussion, however to foreground the topic to other researchers we add two sentences and two references to the background | Lines 86-89 |

Findings:

| Reviewer comment: | Response: | Location: |
| --- | --- | --- |
| I would reconsider use of the word ‘burden’ (line 274) as it is a loaded term | We have now amended this to:  This had the potential to reduce **some of the responsibilities** placed upon them | Line 289 |
| Body work is described as ‘performed directly on the care recipient’s body.’ Could the authors reflect on this language (which makes the person receiving care a passive object) - is it always ‘on’? Or is it sometimes ‘with’? | Thank you for this insight that we had not considered. We have now amended this sentence to:  Participants carried out ‘body work’, **which involved performing tasks involving** the care recipient’s body. | Line 308 |
| The first paragraph on Comfort Work is a bit confusing in places as it moves from talking about ‘Whilst the person lived in their own home…’ to referring to taking the person ‘…out of the care home’. | We have now amended this paragraph to more clearly demarcate where we begin to talk about the comfort work undertaken after the person moved into the care home. For example, we now have a sentence that reads:  Then, **once the person had moved into the care home**, some participants described accompanying the person to activities within the home, or taking the person out of the care home for social activities. | Line 337 |
| I was surprised there wasn’t more about comforting the person in this section - for example at the point of transition to the care home, which can be destabilising and even distressing for some people. If this did not come up in the interviews, I think that would be a finding in itself. | Thank you for raising this issue. We have examples of this within our data, which may have been removed during our own editing process. We have added additional information and a quote to exemplify the work undertaken.  Some participants also provided support and reassurance to the person at times of distress. For example, some participants discussed comforting the care recipient if they experienced distress at the point of a care transition, and a further two participants discussed being called in to the home at times the care recipient experienced distress. On both of these occasions the distress was experienced by a person living with dementia.  *“I was at work and she was having one of these episodes and [the care home staff] rang me and said ‘you need to come now and calm your mum down - she’s shouting”… I got there and sat with her for a good couple of hours.” (BFC14)* | Lines 340 – 349 |
| I like the inclusion of Biography work – that is something that is important but is often overlooked – and I also like the connections made between this and other types of work. | Thank you. | N/A |
| Line 463 refers to an ‘extract above’ describing work to maintain dignity and identity, but that extract seems to be more about how upsetting it is when this work is not done (by care home staff) – it just needs looking at again I think (perhaps they changed the quote when editing?) | Thank you. When looking again, we can see the text was not referring to the extract immediately above, but instead referring to the one above that. In order to be clearer, we have added additional wording and this sentence now reads:  This was **evident in one of the extracts** above**, in which the participant described undertaking biography work to ensure both care home staff and other residents understood the persons current behaviour in the context of their broader biography**. Such work was undertaken to ensure the care recipient’s identity and dignity was maintained. | Lines 493-497 |

Discussion:

| Reviewer comment: | Response: | Location: |
| --- | --- | --- |
| The Discussion section does need some work. For example, cultural and intersectional differences are noted here, but it would be nice to see deeper engagement with these potential nuances. Where the authors write: ‘We use the neutral term ‘body work’, but highlight the ways an individuals’ demographic characteristics and culture can shape their framing of this work.’ there is no reference to the literature. | Please see text at the beginning of this document which explains why we do not feel able to address this comment. | N/A |
| The point about this coordination work being largely present-focused rather than future-focused is important as this has relevance for policy (re people not making advanced care plans etc) and ties in with literature suggesting that people affected by dementia (including families) may not plan for future care needs. There is published evidence on this that could be referenced (see for example Baxter, K., Gridley, K., & Birks, Y. (2025). | Thank you, we have now added in an additional paragraph to this effect. | Lines 642-649 |
| I am not convinced by the explanation of the absence of ‘emotion work’ from the accounts reported. I think the authors need to say more here about what usually comes under the concept of ‘emotion work’ and then explore either where this fits in other aspects of their analysis, or alternatively engage more deeply with why this is missing (which to me is an unexpected finding). Is the difference here something to do with the difference between private and public labour, or could it be an artefact of their data collection (it might not be obvious to the interviewer, or in the interview transcripts, what emotional work the carer is doing behind the scenes, even in that very interview, to manage feelings)? | We have given this issue additional thought and provided further discussion for potential reasons why we did not surface the emotion work undertaken by family carers within our findings | Lines 558-578 |
| Overall, I might have expected more discussion of the dynamic aspects of the findings, which after all are about something that changes over time. | We are unsure whether and how the reviewer would like us to address this comment. | N/A |
| Lines 547-552 imply that carers finding it hard to balance paid work and caring work is a new finding – this is not new, there is a large body of literature on this, including research looking at carers’ decisions to leave employment, and studies evaluating interventions to support their continued employment. I would expect the authors to engage more with the existing literature here (and say what their findings add) rather than just call for more research. | It was not our intention to suggest that this is a new finding. We have added additional sentences to this paragraph to: 1) highlight existing research in this area and 2) call not simply for more research, but for the application of the concept of a care trajectory to enable a more detailed, theoretically informed approach to understanding how people do or do not maintain balance. | Lines 581-591 |
| I am not sure that the reference to more people dying in care settings due to demographic shifts tells the whole story – yes more people are dying in care homes, but I think the trend is also towards more people dying at home – what we are seeing is a policy shift away from dying in hospital (which is also arguably a care setting) (See Bone, A. E., Gomes, B., Etkind, S. N., Verne, J., Murtagh, F. E., Evans, C. J., & Higginson, I. J. (2018). What is the impact of population ageing on the future provision of end-of-life care? Population-based projections of place of death. Palliative medicine, 32(2), 329-336. ) Nevertheless, I agree with the authors that more attention should be paid to the experience of caring for someone dying in a care setting (including where people are in temporary placements). | Whilst we do not disagree with the reviewer, we are unsure whether and how they would like us to address this comment. After careful consideration we have decided not to add the additional references suggested by the author, to prevent having to introduce new topic areas. | N/A |

Implications

| Reviewer comment: | Response: | Location: |
| --- | --- | --- |
| The authors discuss the skills required to do different types of care work – whilst I agree that much of this work is skilled, I do not feel like much evidence was presented about this in the Findings section – perhaps this can be reviewed (and some examples added) if it is going to be mentioned in the Implications? Similarly, they say that their findings support the need for training and education for carers, but the Findings focus on the types of work carers done, not how confident carers feel doing it/how skilled that work is. I have no doubt that training for carers is helpful, but the authors should cite evidence for this (ether form their own interviews, in the Findings, or from the literature in their Discussion). | We have provided an additional quote within the Results section (to provide an example of a family carer undertaking a skilled task) and amended the paragraph in the Discussion (in which we relate our findings to existing research). | Lines 312 – 314 (quotes) /  Lines 652 – 666 (paragraph) |

Strengths and Limitations

| Reviewer comment: | Response: | Location: |
| --- | --- | --- |
| Yes, strong stakeholder engagement is a real strength of this work | Thank you. | N/A |
| I am not convinced by the recommendation for further work line 606 - seems very broad! | We have amended the wording to be more specific.  The study focussed on the work of carers who had experiences of supporting a person with a cognitive impairment who had moved into a care home. Further work could explore **and compare** potential differences in the work **undertaken by different groups** carers, **such as between carers** of people with and without a cognitive impairment and **of carers of** people who do and do not live in a care home. | Lines 685-687 |
| A limitation not listed is the absence of the voice of the people cared for in this research. I understand that the focus is carers and care work, but recognising that there will be multiple perspectives on care work and we only hear one side of a story (that as the authors have said is a social construction) would be helpful | We have acknowledged this in the Discussion section:  Whilst this study aimed to describe the difference forms of work undertaken by family carers, by gathering data from family carers themselves focussed on carers. Further work could gather data from people receiving care. | Lines 680-682 |

Minor things:

| Reviewer comment: | Response: | Location: |
| --- | --- | --- |
| There’s a typo in the abstract (it says ‘wee’ not ‘were’) | Thank you, we have made this change. | Line 23 |
| Another typo on line 238 (first line of Analysis section) - repetition of the word ‘data’) | Thank you, we have made this change. | Line 242 |
| Typo page 439 (says ‘One’ where it should say ‘Once’) | Thank you, we have made this change. | Line 469 |
| 562 – missing word (level ‘of’ care) | Thank you, we have made this change. | Line 619 |
| 582 Typo (‘may changes’ should be ‘may change’) | Thank you, we have made this change. | Line 639 |
