# Supplementary material for: The forms and purpose of work undertaken by family carers of people living in a care home with a cognitive impairment across a care trajectory
Source: PLoS One. 2026 Jun 29;21(6):e0347638. doi: 10.1371/journal.pone.0347638 (PMC13313323; doi:10.1371/journal.pone.0347638)
Supplement: Attachment — Submitted filename: Response to reviewer comments.docx [file pone.0347638.s002.docx]

**Response to reviewer comments**

Manuscript: PONE-D-25-62443R1 - The forms and purpose of work undertaken by family carers of people living in a care home with a cognitive impairment across a care trajectory

| Associate Editor: | Response and revision: | Location: |
| --- | --- | --- |
| The manuscript has been evaluated by one reviewer, who requests additional modifications. Could you please address their comments carefully? | Thank you. We have addressed the comments below. | N/A |

| Reviewer 1 comments: | Response and revision: | Location: |
| --- | --- | --- |
| The [PLOS Data policy](https://eur03.safelinks.protection.outlook.com/?url=http%3A%2F%2Ftrack.editorialmanager.com%2FCL0%2Fhttp%3A%252F%252Fwww.plosone.org%252Fstatic%252Fpolicies.action%2523sharing%2F1%2F010f019e00ee2d92-0e678c1e-a065-42b9-9cee-033b66d6c614-000000%2FVE3ZM_IOclfWkF9quxGPXe7eWRHR9F_317SHAoIHFX0%3D258&data=05%7C02%7Cfawn.harradhyde%40leicester.ac.uk%7C16064437ce584e7f108708deac15b45f%7Caebecd6a31d44b0195ce8274afe853d9%7C0%7C0%7C639137406396007690%7CUnknown%7CTWFpbGZsb3d8eyJFbXB0eU1hcGkiOnRydWUsIlYiOiIwLjAuMDAwMCIsIlAiOiJXaW4zMiIsIkFOIjoiTWFpbCIsIldUIjoyfQ%3D%3D%7C0%7C%7C%7C&sdata=vh9T3Jh2n035tYVSxIC%2FZa1cWmj4QLqa6rxw0eHfelc%3D&reserved=0) requires authors to make all data underlying the findings described in their manuscript fully available without restriction, with rare exception (please refer to the Data Availability Statement in the manuscript PDF file). The data should be provided as part of the manuscript or its supporting information, or deposited to a public repository. For example, in addition to summary statistics, the data points behind means, medians and variance measures should be available. If there are restrictions on publicly sharing data—e.g. participant privacy or use of data from a third party—those must be specified. | We have now deposited all of the data underlying the findings into out institutional repository and this can be accessed at: doi:10.25392/leicester.data.32253444 | N/A |
| Thank you for your detail responses to my comments. I am satisfied with all except the final point on the absence of the voices of people receiving care.  You have added the following sentence to the Discussion (lines 668-9) but I'm not sure this quite makes sense: 'Whilst this study aimed to describe the difference forms of work undertaken by family carers, by gathering data from family carers themselves focussed on carers. Further work could gather data from people receiving care.' Perhaps there are a couple of typos here, but it also doesn't fit well in the paragraph (turns it into a bit of a list) and so I would advise a full read through to ensure that adding sentences like this does not disrupt the flow of the manuscript as a whole before publication. | Thank you. We have re-read the manuscript carefully to identify and address all typos (including the one highlighted here) and to improve the flow. All changes are marked with track changes. | Throughout manuscropt |
