# Supplementary material for: The forms and purpose of work undertaken by family carers of people living in a care home with a cognitive impairment across a care trajectory
Source: PLoS One. 2026 Jun 29;21(6):e0347638. doi: 10.1371/journal.pone.0347638 (PMC13313323; doi:10.1371/journal.pone.0347638)
Supplement: Peer review history

# Peer review: Decision Letter 0

**DOI:** 10.1371/journal.pone.0347638.r001

**Authors:** Alejandro Torrado Pacheco

4 Feb 2026

PONE-D-25-62443

The forms and purpose of work undertaken by family carers of people living in a care home with a cognitive impairment across a care trajectory

PLOS One

Dear Dr. Harrad-Hyde,

Thank you for submitting your manuscript to PLOS ONE. After careful consideration, we feel that it has merit but does not fully meet PLOS ONE’s publication criteria as it currently stands. Therefore, we invite you to submit a revised version of the manuscript that addresses the points raised during the review process.

Please note that we have only been able to secure a single reviewer to assess your manuscript. We are issuing a decision on your manuscript at this point to prevent further delays in the evaluation of your manuscript. Please be aware that the editor who handles your revised manuscript might find it necessary to invite additional reviewers to assess this work once the revised manuscript is submitted. However, we will aim to proceed on the basis of this single review if possible.

The reviewer's comments are available below. They raise a number of points that should be addressed, most notably regarding the discussion of findings. Please note that as per the journal requirements below, citations requested by the reviewer are optional.

Could you please revise the manuscript to carefully address the concerns raised?

Please submit your revised manuscript by Mar 20 2026 11:59PM. If you will need more time than this to complete your revisions, please reply to this message or contact the journal office at plosone@plos.org. When you're ready to submit your revision, log on to https://www.editorialmanager.com/pone/ and select the 'Submissions Needing Revision' folder to locate your manuscript file.

Please include the following items when submitting your revised manuscript:

- A letter that responds to each point raised by the academic editor and reviewer(s). You should upload this letter as a separate file labeled 'Response to Reviewers'.
- A marked-up copy of your manuscript that highlights changes made to the original version. You should upload this as a separate file labeled 'Revised Manuscript with Track Changes'.
- An unmarked version of your revised paper without tracked changes. You should upload this as a separate file labeled 'Manuscript'.

If you would like to make changes to your financial disclosure, please include your updated statement in your cover letter. Guidelines for resubmitting your figure files are available below the reviewer comments at the end of this letter.

If applicable, we recommend that you deposit your laboratory protocols in protocols.io to enhance the reproducibility of your results. Protocols.io assigns your protocol its own identifier (DOI) so that it can be cited independently in the future. For instructions see: https://journals.plos.org/plosone/s/submission-guidelines#loc-laboratory-protocols. Additionally, PLOS ONE offers an option for publishing peer-reviewed Lab Protocol articles, which describe protocols hosted on protocols.io. Read more information on sharing protocols at https://plos.org/protocols?utm_medium=editorial-email&utm_source=authorletters&utm_campaign=protocols.

We look forward to receiving your revised manuscript.

Kind regards,

Alejandro Torrado Pacheco, PhD

Staff Editor

PLOS One

Journal Requirements:

When submitting your revision, we need you to address these additional requirements.

1. Please ensure that your manuscript meets PLOS ONE's style requirements, including those for file naming. The PLOS ONE style templates can be found at

https://journals.plos.org/plosone/s/file?id=wjVg/PLOSOne_formatting_sample_main_body.pdf and

https://journals.plos.org/plosone/s/file?id=ba62/PLOSOne_formatting_sample_title_authors_affiliations.pdf

2. Thank you for stating the following in the Acknowledgments Section of your manuscript:

“This work was supported by a grant from LOROS Centre for Excellence. The views expressed are those of the authors and not necessarily those of LOROS Centre for Excellence.”

We note that you have provided funding information that is not currently declared in your Funding Statement. However, funding information should not appear in the Acknowledgments section or other areas of your manuscript. We will only publish funding information present in the Funding Statement section of the online submission form.

Please remove any funding-related text from the manuscript and let us know how you would like to update your Funding Statement. Currently, your Funding Statement reads as follows:

“This work was supported by a grant from LOROS Centre for Excellence. The views expressed are those of the authors and not necessarily those of LOROS Centre for Excellence.”

Please include your amended statements within your cover letter; we will change the online submission form on your behalf.

3. We note that the grant information you provided in the ‘Funding Information’ and ‘Financial Disclosure’ sections do not match.

When you resubmit, please ensure that you provide the correct grant numbers for the awards you received for your study in the ‘Funding Information’ section.

4. Thank you for stating the following financial disclosure:

“This work was supported by a grant from LOROS Centre for Excellence. The views expressed are those of the authors and not necessarily those of LOROS Centre for Excellence.”

Please state what role the funders took in the study.  If the funders had no role, please state: "The funders had no role in study design, data collection and analysis, decision to publish, or preparation of the manuscript."

If this statement is not correct you must amend it as needed.

Please include this amended Role of Funder statement in your cover letter; we will change the online submission form on your behalf.

5. Thank you for stating the following financial disclosure:

“This work was supported by a grant from LOROS Centre for Excellence. The views expressed are those of the authors and not necessarily those of LOROS Centre for Excellence.”

We note that one or more of the authors is affiliated with the funding organization, indicating the funder may have had some role in the design, data collection, analysis or preparation of your manuscript for publication; in other words, the funder played an indirect role through the participation of the co-authors. If the funding organization did not play a role in the study design, data collection and analysis, decision to publish, or preparation of the manuscript and only provided financial support in the form of authors' salaries and/or research materials, please do the following:

1. Review your statements relating to the author contributions, and ensure you have specifically and accurately indicated the role(s) that these authors had in your study. These amendments should be made in the online form.

2. Confirm in your cover letter that you agree with the following statement, and we will change the online submission form on your behalf:

“The funder provided support in the form of salaries for authors [insert relevant initials], but did not have any additional role in the study design, data collection and analysis, decision to publish, or preparation of the manuscript. The specific roles of these authors are articulated in the ‘author contributions’ section.

6. In the online submission form you indicate that your data is not available for proprietary reasons and have provided a contact point for accessing this data. Please note that your current contact point is a co-author on this manuscript. According to our Data Policy, the contact point must not be an author on the manuscript and must be an institutional contact, ideally not an individual. Please revise your data statement to a non-author institutional point of contact, such as a data access or ethics committee, and send this to us via return email. Please also include contact information for the third party organization, and please include the full citation of where the data can be found.

7. Please include a copy of Table 1 which you refer to in your text on page 10.

8. If the reviewer comments include a recommendation to cite specific previously published works, please review and evaluate these publications to determine whether they are relevant and should be cited. There is no requirement to cite these works unless the editor has indicated otherwise.

[Note: HTML markup is below. Please do not edit.]

Reviewers' comments:

Reviewer's Responses to Questions

**Comments to the Author**

1. Is the manuscript technically sound, and do the data support the conclusions?

The manuscript must describe a technically sound piece of scientific research with data that supports the conclusions. Experiments must have been conducted rigorously, with appropriate controls, replication, and sample sizes. The conclusions must be drawn appropriately based on the data presented.

Reviewer #1: Yes

**********

2. Has the statistical analysis been performed appropriately and rigorously?

Reviewer #1: N/A

**********

3. Have the authors made all data underlying the findings in their manuscript fully available?

The PLOS Data policy requires authors to make all data underlying the findings described in their manuscript fully available without restriction, with rare exception (please refer to the Data Availability Statement in the manuscript PDF file). The data should be provided as part of the manuscript or its supporting information, or deposited to a public repository. For example, in addition to summary statistics, the data points behind means, medians and variance measures should be available. If there are restrictions on publicly sharing data—e.g. participant privacy or use of data from a third party—those must be specified.

Reviewer #1: No

**********

4. Is the manuscript presented in an intelligible fashion and written in standard English?

PLOS ONE does not copyedit accepted manuscripts, so the language in submitted articles must be clear, correct, and unambiguous. Any typographical or grammatical errors should be corrected at revision, so please note any specific errors here.

Reviewer #1: Yes

**********

5. Review Comments to the Author

Please use the space provided to explain your answers to the questions above. You may also include additional comments for the author, including concerns about dual publication, research ethics, or publication ethics. (Please upload your review as an attachment if it exceeds 20,000 characters)

Reviewer #1: Overview:

I enjoyed reading this paper and think in general it is well written and will be a useful addition to the literature. The paper starts with a good overview of the background and theory to this topic and the aims and rationale are clearly articulated. The focus on care work throughout the trajectory is welcome and it makes sense to look at this specifically for carers of people with cognitive impairment as the experience can differ for this group than, for example, carers for older people with physical impairment of frailty alone.

I like that the authors set out their position early on, both philosophically, and in terms of their lived experience, and a particular strength is their long-term engagement with a wider group of stakeholders. I did think that the authors’ reference to ‘accuracy’ (line 199) is perhaps inconsistent with their social constructivist philosophy – do they mean here that they checked back with stakeholders for their agreement on the account recorded?

The methods are well described, in particular the approach to recruitment and purposive sampling. I personally feel it is a shame that these were just one-off interviews, as longitudinal interviews would have given more opportunity to explore change over time, but focusing on carers’ accounts on trajectories is still valuable. This could perhaps be picked up in the limitation section.

I would like to see a little more detail in the Analysis section. In particular, how did the constructivist approach influence analysis? How did you move from codes to themes? Were stakeholders involved in this process?

I do not seem to have access to the demographics table, but it is mentioned in the limitations section that most participants were White British. However, the role of culture in care work is raised a few times in the Findings and I think this was an omission from the Background section (for example, there was nothing about how carers in different cultures experience care work). Similarly, the influence of culture on experiences of care could be returned to in the Discussion section.

Points to consider from the Findings section:

• I would reconsider use of the word ‘burden’ (line 274) as it is a loaded term

• Body work is described as ‘performed directly on the care recipient’s body.’ I appreciate that this is grounded in the literature/existing theory, but could the authors reflect on this language (which makes the person receiving care a passive object) - is it always ‘on’? Or is it sometimes ‘with’?

• The first paragraph on Comfort Work is a bit confusing in places as it moves from talking about ‘Whilst the person lived in their own home…’ to referring to taking the person ‘…out of the care home’.

• I was surprised there wasn’t more about comforting the person in this section - for example at the point of transition to the care home, which can be destabilising and even distressing for some people. If this did not come up in the interviews, I think that would be a finding in itself.

• I like the inclusion of Biography work – that is something that is important but is often overlooked – and I also like the connections made between this and other types of work.

• Line 463 refers to an ‘extract above’ describing work to maintain dignity and identity, but that extract seems to be more about how upsetting it is when this work is not done (by care home staff) – it just needs looking at again I think (perhaps they changed the quote when editing?)

Discussion:

For me, the Discussion section does need some work. For example, cultural and intersectional differences are noted here, but it would be nice to see deeper engagement with these potential nuances. Where the authors write: ‘We use the neutral term ‘body work’, but highlight the ways an individuals’ demographic characteristics and culture can shape their framing of this work.’ there is no reference to the literature, but I find it hard to believe that there isn’t any (and if there really isn’t then this itself is a point worth raising).

I agree that ‘coordination’ is a useful addition (potentially more relevant in this context than articulation work, although it may incorporate this.) The point about this coordination work being largely present-focused rather than future-focused is important as this has relevance for policy (re people not making advanced care plans etc) and ties in with literature suggesting that people affected by dementia (including families) may not plan for future care needs. There is published evidence on this that could be referenced (see for example Baxter, K., Gridley, K., & Birks, Y. (2025). The role of uncertainty in planning for self-funded social care for older people with a diagnosis of dementia. Ageing & Society, 45(3), 514-534. ).

On the other hand, I am not convinced by the explanation of the absence of ‘emotion work’ from the accounts reported. I think the authors need to say more here about what usually comes under the concept of ‘emotion work’ and then explore either where this fits in other aspects of their analysis, or alternatively engage more deeply with why this is missing (which to me is an unexpected finding). Is the difference here something to do with the difference between private and public labour, or could it be an artefact of their data collection (it might not be obvious to the interviewer, or in the interview transcripts, what emotional work the carer is doing behind the scenes, even in that very interview, to manage feelings)?

Overall, I might have expected more discussion of the dynamic aspects of the findings, which after all are about something that changes over time.

Lines 547-552 imply that carers finding it hard to balance paid work and caring work is a new finding – this is not new, there is a large body of literature on this, including research looking at carers’ decisions to leave employment, and studies evaluating interventions to support their continued employment. I would expect the authors to engage more with the existing literature here (and say what their findings add) rather than just call for more research.

I am not sure that the reference to more people dying in care settings due to demographic shifts tells the whole story – yes more people are dying in care homes, but I think the trend is also towards more people dying at home – what we are seeing is a policy shift away from dying in hospital (which is also arguably a care setting) (See Bone, A. E., Gomes, B., Etkind, S. N., Verne, J., Murtagh, F. E., Evans, C. J., & Higginson, I. J. (2018). What is the impact of population ageing on the future provision of end-of-life care? Population-based projections of place of death. Palliative medicine, 32(2), 329-336. ) Nevertheless, I agree with the authors that more attention should be paid to the experience of caring for someone dying in a care setting (including where people are in temporary placements).

Implications

The authors discuss the skills required to do different types of care work – whilst I agree that much of this work is skilled, I do not feel like much evidence was presented about this in the Findings section – perhaps this can be reviewed (and some examples added) if it is going to be mentioned in the Implications? Similarly, they say that their findings support the need for training and education for carers, but the Findings focus on the types of work carers done, not how confident carers feel doing it/how skilled that work is. I have no doubt that training for carers is helpful, but the authors should cite evidence for this (ether form their own interviews, in the Findings, or from the literature in their Discussion).

Strengths and Limitations

Yes, strong stakeholder engagement is a real strength of this work.

I am not convinced by the recommendation for further work line 606 - seems very broad!

A limitation not listed is the absence of the voice of the people cared for in this research. I understand that the focus is carers and care work, but recognising that there will be multiple perspectives on care work and we only hear one side of a story (that as the authors have said is a social construction) would be helpful.

Minor things:

• There’s a typo in the abstract (it says ‘wee’ not ‘were’)

• Another typo on line 238 (first line of Analysis section) - repetition of the word ‘data’)

• Typo page 439 (says ‘One’ where it should say ‘Once’)

• 562 – missing word (level ‘of’ care)

• 582 Typo (‘may changes’ should be ‘may change’)

Thank you for the opportunity to review this interesting paper. I would recommend publication with minor revisions.

**********

6. PLOS authors have the option to publish the peer review history of their article (what does this mean?). If published, this will include your full peer review and any attached files.

If you choose “no”, your identity will remain anonymous but your review may still be made public.

**Do you want your identity to be public for this peer review?**  For information about this choice, including consent withdrawal, please see our Privacy Policy.

Reviewer #1: No

**********

[NOTE: If reviewer comments were submitted as an attachment file, they will be attached to this email and accessible via the submission site. Please log into your account, locate the manuscript record, and check for the action link "View Attachments". If this link does not appear, there are no attachment files.]

To ensure your figures meet our technical requirements, please review our figure guidelines: https://journals.plos.org/plosone/s/figures

You may also use PLOS’s free figure tool, NAAS, to help you prepare publication quality figures: https://journals.plos.org/plosone/s/figures#loc-tools-for-figure-preparation.

NAAS will assess whether your figures meet our technical requirements by comparing each figure against our figure specifications.

# Author comment: Author response to Decision Letter 1

**DOI:** 10.1371/journal.pone.0347638.r002

9 Mar 2026

We would also like to thank our reviewer for taking the time to read and review our paper. We are grateful that they acknowledge the paper’s strengths, alongside areas which could be further improved. We outline how we have addressed each of these comments on a point-by-point basis in the 'response to reviewers' document uploaded as part of this re-submission.

Attachment Submitted filename: Response to Reviewers.docx

# Peer review: Decision Letter 1

**DOI:** 10.1371/journal.pone.0347638.r003

**Authors:** Alejandro Torrado Pacheco

7 May 2026

PONE-D-25-62443R1

The forms and purpose of work undertaken by family carers of people living in a care home with a cognitive impairment across a care trajectory

PLOS One

Dear Dr. Harrad-Hyde,

Thank you for submitting your manuscript to PLOS ONE. After careful consideration, we feel that it has merit but does not fully meet PLOS ONE’s publication criteria as it currently stands. Therefore, we invite you to submit a revised version of the manuscript that addresses the points raised during the review process.

Please submit your revised manuscript by Jun 19 2026 11:59PM. If you will need more time than this to complete your revisions, please reply to this message or contact the journal office at plosone@plos.org. When you're ready to submit your revision, log on to https://www.editorialmanager.com/pone/ and select the 'Submissions Needing Revision' folder to locate your manuscript file.

Please include the following items when submitting your revised manuscript:

- A letter that responds to each point raised by the academic editor and reviewer(s). You should upload this letter as a separate file labeled 'Response to Reviewers'.
- A marked-up copy of your manuscript that highlights changes made to the original version. You should upload this as a separate file labeled 'Revised Manuscript with Track Changes'.
- An unmarked version of your revised paper without tracked changes. You should upload this as a separate file labeled 'Manuscript'.

If you would like to make changes to your financial disclosure, please include your updated statement in your cover letter. Guidelines for resubmitting your figure files are available below the reviewer comments at the end of this letter.

If applicable, we recommend that you deposit your laboratory protocols in protocols.io to enhance the reproducibility of your results. Protocols.io assigns your protocol its own identifier (DOI) so that it can be cited independently in the future. For instructions see: https://journals.plos.org/plosone/s/submission-guidelines#loc-laboratory-protocols. Additionally, PLOS ONE offers an option for publishing peer-reviewed Lab Protocol articles, which describe protocols hosted on protocols.io. Read more information on sharing protocols at https://plos.org/protocols?utm_medium=editorial-email&utm_source=authorletters&utm_campaign=protocols.

As the corresponding author, your ORCID iD is verified in the submission system and will appear in the published article. PLOS supports the use of ORCID, and we encourage all coauthors to register for an ORCID iD and use it as well. Please encourage your coauthors to verify their ORCID iD within the submission system before final acceptance, as unverified ORCID iDs will not appear in the published article. *Only*  the individual author can complete the verification step; PLOS staff *cannot*  verify ORCID iDs on behalf of authors.

We look forward to receiving your revised manuscript.

Kind regards,

Alejandro Torrado Pacheco, PhD

Associate Editor

PLOS One

Journal Requirements:

1. If the reviewer comments include a recommendation to cite specific previously published works, please review and evaluate these publications to determine whether they are relevant and should be cited. There is no requirement to cite these works unless the editor has indicated otherwise.

Additional Editor Comments:

The manuscript has been evaluated by one reviewer, who requests additional modifications. Could you please address their comments carefully?

[Note: HTML markup is below. Please do not edit.]

Reviewer's Responses to Questions

**Comments to the Author**

1. If the authors have adequately addressed your comments raised in a previous round of review and you feel that this manuscript is now acceptable for publication, you may indicate that here to bypass the “Comments to the Author” section, enter your conflict of interest statement in the “Confidential to Editor” section, and submit your "Accept" recommendation.

Reviewer #1: All comments have been addressed

**********

2. Is the manuscript technically sound, and do the data support the conclusions?

The manuscript must describe a technically sound piece of scientific research with data that supports the conclusions. Experiments must have been conducted rigorously, with appropriate controls, replication, and sample sizes. The conclusions must be drawn appropriately based on the data presented.

Reviewer #1: Yes

**********

3. Has the statistical analysis been performed appropriately and rigorously?

Reviewer #1: N/A

**********

4. Have the authors made all data underlying the findings in their manuscript fully available?

The PLOS Data policy requires authors to make all data underlying the findings described in their manuscript fully available without restriction, with rare exception (please refer to the Data Availability Statement in the manuscript PDF file). The data should be provided as part of the manuscript or its supporting information, or deposited to a public repository. For example, in addition to summary statistics, the data points behind means, medians and variance measures should be available. If there are restrictions on publicly sharing data—e.g. participant privacy or use of data from a third party—those must be specified.

Reviewer #1: No

**********

5. Is the manuscript presented in an intelligible fashion and written in standard English?

PLOS ONE does not copyedit accepted manuscripts, so the language in submitted articles must be clear, correct, and unambiguous. Any typographical or grammatical errors should be corrected at revision, so please note any specific errors here.

Reviewer #1: Yes

**********

6. Review Comments to the Author

Please use the space provided to explain your answers to the questions above. You may also include additional comments for the author, including concerns about dual publication, research ethics, or publication ethics. (Please upload your review as an attachment if it exceeds 20,000 characters)

Reviewer #1: Thank you for your detail responses to my comments. I am satisfied with all except the final point on the absence of the voices of people receiving care. You have added the following sentence to the Discussion (lines 668-9) but I'm not sure this quite makes sense: 'Whilst this study aimed to describe the difference forms of work undertaken by family carers, by gathering data from family carers themselves focussed on carers. Further work could gather data from people receiving care.' Perhaps there are a couple of typos here, but it also doesn't fit well in the paragraph (turns it into a bit of a list) and so I would advise a full read through to ensure that adding sentences like this does not disrupt the flow of the manuscript as a whole before publication.

**********

7. PLOS authors have the option to publish the peer review history of their article (what does this mean?). If published, this will include your full peer review and any attached files.

If you choose “no”, your identity will remain anonymous but your review may still be made public.

**Do you want your identity to be public for this peer review?**  For information about this choice, including consent withdrawal, please see our Privacy Policy.

Reviewer #1: No

**********

[NOTE: If reviewer comments were submitted as an attachment file, they will be attached to this email and accessible via the submission site. Please log into your account, locate the manuscript record, and check for the action link "View Attachments". If this link does not appear, there are no attachment files.]

To ensure your figures meet our technical requirements, please review our figure guidelines: https://journals.plos.org/plosone/s/figures

You may also use PLOS’s free figure tool, NAAS, to help you prepare publication quality figures: https://journals.plos.org/plosone/s/figures#loc-tools-for-figure-preparation.

NAAS will assess whether your figures meet our technical requirements by comparing each figure against our figure specifications.

# Author comment: Author response to Decision Letter 2

**DOI:** 10.1371/journal.pone.0347638.r004

12 May 2026

We have addressed the two issues raised during the review process. First, we have made our data underlying our findings available to others via a DOI. Second, we have searched the manuscript for typos and addressed these, with a focus on improving flow. All of these changes are visible via track changes.

We have attached a Response to Reviewers document to this submission.

Attachment Submitted filename: Response to reviewer comments.docx

# Peer review: Decision Letter 2

**DOI:** 10.1371/journal.pone.0347638.r005

**Authors:** Gursharan Singh

5 Jun 2026

The forms and purpose of work undertaken by family carers of people living in a care home with a cognitive impairment across a care trajectory

PONE-D-25-62443R2

Dear Dr. Harrad-Hyde,

We’re pleased to inform you that your manuscript has been judged scientifically suitable for publication and will be formally accepted for publication once it meets all outstanding technical requirements.

Within one week, you’ll receive an e-mail detailing the required amendments. When these have been addressed, you’ll receive a formal acceptance letter and your manuscript will be scheduled for publication.

An invoice will be generated when your article is formally accepted. Please note, if your institution has a publishing partnership with PLOS and your article meets the relevant criteria, all or part of your publication costs will be covered. Please make sure your user information is up-to-date by logging into Editorial Manager at Editorial Manager® and clicking the ‘Update My Information' link at the top of the page. For questions related to billing, please contact billing support.

If your institution or institutions have a press office, please notify them about your upcoming paper to help maximize its impact. If they’ll be preparing press materials, please inform our press team as soon as possible -- no later than 48 hours after receiving the formal acceptance. Your manuscript will remain under strict press embargo until 2 pm Eastern Time on the date of publication. For more information, please contact onepress@plos.org.

Kind regards,

Gursharan K Singh, PhD

Academic Editor

PLOS One

Additional Editor Comments (optional):

Reviewers' comments:

Reviewer's Responses to Questions

**Comments to the Author**

1. If the authors have adequately addressed your comments raised in a previous round of review and you feel that this manuscript is now acceptable for publication, you may indicate that here to bypass the “Comments to the Author” section, enter your conflict of interest statement in the “Confidential to Editor” section, and submit your "Accept" recommendation.

Reviewer #1: All comments have been addressed

**********

2. Is the manuscript technically sound, and do the data support the conclusions?

The manuscript must describe a technically sound piece of scientific research with data that supports the conclusions. Experiments must have been conducted rigorously, with appropriate controls, replication, and sample sizes. The conclusions must be drawn appropriately based on the data presented.

Reviewer #1: Yes

**********

3. Has the statistical analysis been performed appropriately and rigorously?

Reviewer #1: N/A

**********

4. Have the authors made all data underlying the findings in their manuscript fully available?

The PLOS Data policy requires authors to make all data underlying the findings described in their manuscript fully available without restriction, with rare exception (please refer to the Data Availability Statement in the manuscript PDF file). The data should be provided as part of the manuscript or its supporting information, or deposited to a public repository. For example, in addition to summary statistics, the data points behind means, medians and variance measures should be available. If there are restrictions on publicly sharing data—e.g. participant privacy or use of data from a third party—those must be specified.

Reviewer #1: Yes

**********

5. Is the manuscript presented in an intelligible fashion and written in standard English?

PLOS ONE does not copyedit accepted manuscripts, so the language in submitted articles must be clear, correct, and unambiguous. Any typographical or grammatical errors should be corrected at revision, so please note any specific errors here.

Reviewer #1: Yes

**********

6. Review Comments to the Author

Please use the space provided to explain your answers to the questions above. You may also include additional comments for the author, including concerns about dual publication, research ethics, or publication ethics. (Please upload your review as an attachment if it exceeds 20,000 characters)

Reviewer #1: Thank you for addressing the final comments - I am satisfied that this paper is suitable for publication (there is a typo line 688 but that's very easily fixable).

**********

7. PLOS authors have the option to publish the peer review history of their article (what does this mean?). If published, this will include your full peer review and any attached files.

If you choose “no”, your identity will remain anonymous but your review may still be made public.

**Do you want your identity to be public for this peer review?**  For information about this choice, including consent withdrawal, please see our Privacy Policy.

Reviewer #1: No

**********

# Editor report: Acceptance letter

**DOI:** 10.1371/journal.pone.0347638.r006

**Authors:** Gursharan Singh

PONE-D-25-62443R2

PLOS One

Dear Dr. Harrad-Hyde,

I'm pleased to inform you that your manuscript has been deemed suitable for publication in PLOS One. Congratulations! Your manuscript is now being handed over to our production team.

At this stage, our production department will prepare your paper for publication. This includes ensuring the following:

* All references, tables, and figures are properly cited

* All relevant supporting information is included in the manuscript submission,

* There are no issues that prevent the paper from being properly typeset

You will receive further instructions from the production team, including instructions on how to review your proof when it is ready. Please keep in mind that we are working through a large volume of accepted articles, so please give us a few days to review your paper and let you know the next and final steps.

Lastly, if your institution or institutions have a press office, please let them know about your upcoming paper now to help maximize its impact. If they'll be preparing press materials, please inform our press team within the next 48 hours. Your manuscript will remain under strict press embargo until 2 pm Eastern Time on the date of publication. For more information, please contact onepress@plos.org.

You will receive an invoice from PLOS for your publication fee after your manuscript has reached the completed accept phase. If you receive an email requesting payment before acceptance or for any other service, this may be a phishing scheme. Learn how to identify phishing emails and protect your accounts at https://explore.plos.org/phishing.

If we can help with anything else, please email us at customercare@plos.org.

Thank you for submitting your work to PLOS ONE and supporting open access.

Kind regards,

PLOS ONE Editorial Office Staff

on behalf of

Dr. Gursharan K Singh

Academic Editor

PLOS One
